# Optimization of single plate-serial dilution spotting (SP-SDS) with sample anchoring as an assured method for bacterial and yeast cfu enumeration and single colony isolation from diverse samples

**DOI:** 10.1016/j.btre.2015.08.003

**Published:** 2015-08-20

**Authors:** Pious Thomas, Aparna C. Sekhar, Reshmi Upreti, Mohammad M. Mujawar, Sadiq S. Pasha

**Affiliations:** Division of Biotechnology, Indian Institute of Horticultural Research, Hessaraghatta Lake, Bangalore 560089, India

**Keywords:** cfu, colony forming units, CNA, cetrimide- nalixic acid- agar, OD, optical density, NA, nutrient agar, NB, nutrient broth, PDA, potato dextrose agar, PP, polypropylene bag, PS, peptone-salt, SATS, spotting- and- tilt- spreading, SP-SDS, single plate-serial dilution spotting, tmtc, too many to count, Agricultural biotechnology, cfu Estimation, Environmental biotechnology, Food microbiology, Pour-plating, Spread-plating

## Abstract

We propose a simple technique for bacterial and yeast cfu estimations from diverse samples with no prior idea of viable counts, designated as single plate-serial dilution spotting (SP-SDS) with the prime recommendation of sample anchoring (10^0^ stocks). For pure cultures, serial dilutions were prepared from 0.1 OD (10^0^) stock and 20 μl aliquots of six dilutions (10^1^–10^6^) were applied as 10–15 micro-drops in six sectors over agar-gelled medium in 9-cm plates. For liquid samples 10^0^–10^5^ dilutions, and for colloidal suspensions and solid samples (10% w/v), 10^1^–10^6^ dilutions were used. Following incubation, at least one dilution level yielded 6–60 cfu per sector comparable to the standard method involving 100 μl samples. Tested on diverse bacteria, composite samples and *Saccharomyces cerevisiae*, SP-SDS offered wider applicability over alternative methods like drop-plating and track-dilution for cfu estimation, single colony isolation and culture purity testing, particularly suiting low resource settings.

## Highlights

• SP-SDS forms a simple tool for bacterial cfu estimation for samples with unknown cfu.
• Prime recommendation of anchoring specimens to fixed initial OD or a standard base.
• Six serial dilutions of 20 μl each applied per 9-cm plate followed by manual counting.
• Suits pure and mixed bacterial stocks, spores, yeasts and composite samples.
• Superior to alternate techniques like track-dilution, drop-plating or drop-spotting.

## Graphical abstract

**Figure:**
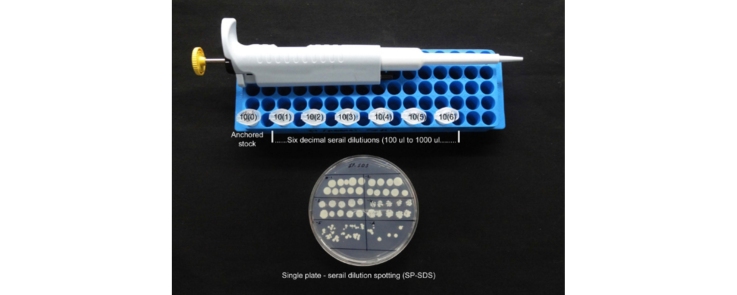

## 1 Introduction

Estimation of colony forming units (cfu) through serial dilution plating on a nutrient medium forms the most widely accepted method for monitoring cultivable bacteria and yeasts in different spheres of microbiology [14], [27]. Cultivation-based methods being simple to practice, command enormous significance and applications in bacteriology. This holds good in spite of the emergence of molecular techniques such as fluorescent in situ hybridization, real-time quantitative PCR, flow cytometry, etc., which although provide a precise account of metabolically active cells [3], [20] demand much expertise and resources. Further, cfu-based techniques provide information on the most abundant populations among the cultivable community [4], [17]. Viable colony counts also form essential tools in biotechnology such as gene cloning, surveillance of genetically modified organisms, assessing bioremediation effects, testing novel anti-microbials, etc. besides serving as standards during molecular investigations.

Spread-plating and pour-plating form the standard approaches for bacterial and yeast cfu estimations [8], [9], [14], [15]. Spread-plating offers several advantages over pour-plating such as more flexibility in handling, less interfering effects on temperature sensitive organisms, the avoidance of aerobic organisms getting trapped inside agar medium, the surface enumeration of cfu and the easy selection of distinct colony types [7], [15], [27]. Here, the bacterial sample is applied over agar-gelled nutrient medium with the help of a glass, plastic or steel spreader where the spreader is generally considered a mere tool to distribute the inoculum over the agar surface [5], [15], [28]. We have documented that the inoculum-spreader employed during standard spread-plating could impart significant injury to bacterial cells and affect the cfu depending on the extent of its usage on the agar surface [23]. This was demonstrated in comparison with the alternate approach that did not involve the use of spreader, namely, spotting- and- tilt- spreading (SATS). Any spreader movement on agar surface subsequent to the exhaustion of free moisture proved detrimental to the bacterial cells further influenced by the operator practices and moisture levels in the medium. The physical impaction effects on vegetative cells varied between different organisms governed by the cell characteristics of the bacterium with Gram-negative organisms being more vulnerable than Gram-positive bacteria, cocci less susceptible than rods and more risk to larger cells than smaller cells [25]. The physical impaction effect also applied to the supposedly hardy spores of *Bacillus* spp. which seemed comparable to glass globules that crumble under physical pressure [26]. Thus, the spreader-independent SATS approach proved to be a simpler and safer alternative to spread-plating for bacterial cfu estimations with several other advantages [23], [25], [26].

Generally 25–250 or 30–300 colonies per agar plate (100 μl sample) are prescribed as the acceptable cfu for accurate counting [14], [23], [25], [27]. When there is no clear indication of the dilution level that yields this cfu range, several plates representing different dilutions and replications need to be employed leading to considerable wastage of time, manpower and material resources [2], [6], [13]. This applies invariably to pure bacterial cultures, water, food, soil and various environmental and biotechnological specimens. As we found that inoculum-spreader was wholly dispensable, accommodating multiple dilutions in a plate was considered. Similar attempts in the past included drop-plating [2], [6], [16], track-dilution [13] and drop-spotting with digital imaging [19], but these studies used pure bacterial cultures that yielded confined colony growths. The situation is different when the samples involve fast growing organisms, mixture of different bacteria varying in growth rates or colony characteristics, and with food and environmental samples. The present studies were undertaken to optimize a simple and resource saving method for bacterial cfu estimations that allows the accommodation of multiple dilutions in a plate and to test the feasibility of the technique across diverse samples including pure bacterial and yeast cultures and composite samples.

## 2 Materials and methods

### 2.1 Bacterial and yeast cultures and composite samples

Pure cultures of bacteria belonging to different phylogenetic groups varying in Gram reaction, cell characteristics and sporulation potential were used towards optimizing the single plate-serial dilution spotting (SP-SDS) technique employing spotting- and- tilt- spreading (SATS) [23], [25], as the standard procedure. The organisms included *Enterobacter cloacae*, *Escherichia coli*, *Acinetobacter junii* (Proteobacteria), *Bacillus pumilus, Bacillus subtilis, Bacillus thuringiensis, Staphylococcus epidermidis, Staphylococcus haemolyticus* (Firmicutes) and *Microbacterium esteraromaticum* (Actinobacterium) described elsewhere [25]. *E. cloacae* was used as the primary candidate for protocol optimization followed by *B. pumilus*. One strain of ascosporogeneous wine yeast (*Saccharomyces cerevisiae*) was used in this study employing potato dextrose agar (PDA). Different composite samples representing public health, food, environmental, agricultural, clinical and biotechnological settings described below were also tested for bacterial or yeast cfu. Additionally, an endophytic bacterial strain of *Pseudomonas aeruginosa* from banana [18] that could be monitored distinctly from other organisms on cetrimide- nalidixic acid- agar (CNA) selective medium [22] was used as a representative of clinical specimens and genetically modified organisms. Unless mentioned differently, overnight nutrient agar (NA)/nutrient broth (NB) derived (18–24 h) cultures were used in all studies involving pure bacterial cultures except for spores.

### 2.2 Nutrient media

Nutrient agar sourced from M/s HiMedia Biosciences (Mumbai, India) formed the standard bacteriological medium while the other media formulations mentioned later were employed for specific organisms/samples and also to test the applicability across different media. Unless mentioned differently, NA/fresh PDA prepared in pre-sterilized disposable Petri-dishes on the same day about 2 h post-pouring (referred to as fresh plates) or that prepared on the previous day and incubated overnight at 37 °C after sealing in polypropylene (PP) bags were used in all trials. The nutrient plates used in a specific trial belonged to the same batch of preparation unless mentioned differently.

### 2.3 SP-SDS and SATS procedures

For pure bacterial and yeast cultures, a uniform cell suspension was prepared by dispersing the overnight colony growths from agar plates, or NB culture after one spin-wash in sterile water in the case of *Bacillus* spp. After allowing any cell clumps to settle down, the clear upper part was transferred to a fresh tube. The optical density (OD) was determined at 600 nm employing a 1:10 diluted stock in a uv/vis spectrophotometer (Genesis 10 UV, Thermo Scientific, MA, USA) based on which the ‘anchored stock’ of 0.1 OD (10^0^) was prepared. Decimal serial dilutions (100–1000 μl) of 10^1^–10^6^ or 10^7^ were prepared from the 10^0^ stock in 1.5 ml tubes with 4–5 repeated flushing and changing of tips (see movie: https://youtu.be/LEqmWmBVIpA). For preparing the stock and serial dilutions, filter-sterilized distilled water (FDW) aliquoted and stored at −20 °C was preferred unless the water was freshly autoclaved. This was essential to avoid the chances of any hardy autoclaving defying spores multiplying during the post-autoclaving storage [21]. Spore preparations and dilutions were made from 7-day old NA plates in 50% ethanol as described elsewhere to avoid their germination [24]. For water and clear liquid specimens, the direct sample formed the 10^0^ anchored stocks. Thick and colloidal suspensions such as milk and fruit juice were used directly or after adjusting OD600 nm to 1.0 or 10 while for solid specimens (food, soil) a suspension prepared in water at 1.0 g sample per 10 ml formed the 10^0^ stock. In this study, our emphasis was on cfu enumeration technique rather than sampling methods for which the accepted standard procedures prescribed were to be adhered (e.g. [8], [9], [10], [11], [12], [13], [14]).

**Fig. 1:**
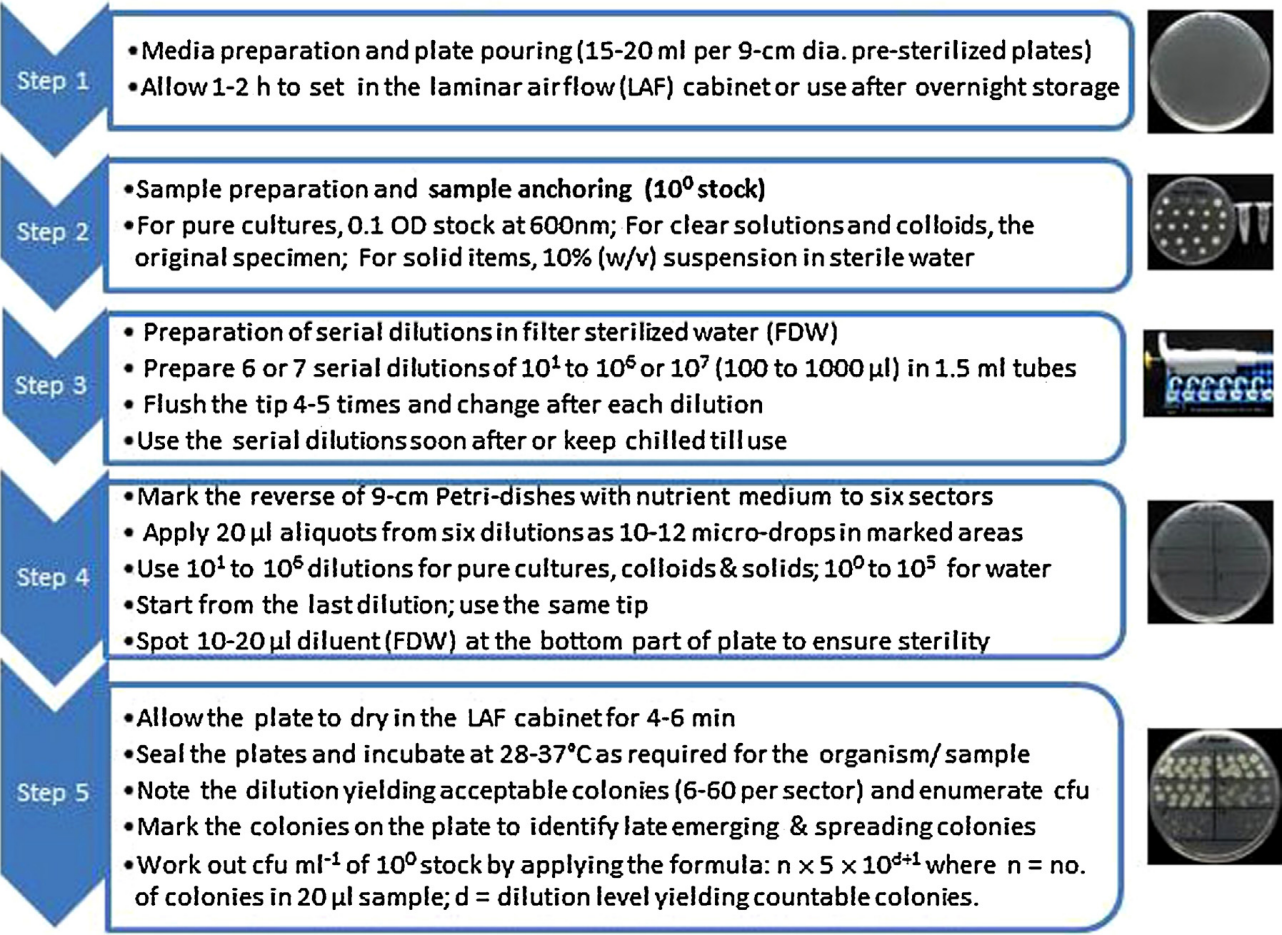
An illustration of single plate-serial dilution spotting (SP-SDS) technique for pure bacterial / yeast cultures and composite samples.

To execute SP-SDS, the reverse of the 9-cm Petri-dishes containing surface dry agar media were drawn to six sectors with the marking of first and last dilution sectors for clear identification. Using a calibrated micropipette, 20 μl aliquots from selected six dilutions were applied as 10–12 micro-drops in these demarcated areas (Fig. 1). During sample spotting, the same tip was used starting with the lowest dilution. Care was exercised to avoid tip marks on the medium during sample application not to mistake them for cfu. The sterility of the diluent was ensured by spotting 20 μl at the bottom part of the plate. The plates were exposed in the laminar air-flow (LAF) cabinet for the droplets to dry off (8–10 min for fresh plates and 3–4 min for pre-prepared surface-dry plates), sealed in polypropylene (PP) covers and incubated inverted at 28–37 °C as required for specific organisms. For SATS, 100 μl of different dilutions were applied as 20–25 micro-drops per plate and spread on agar surface by mere tilting or gentle twirling of plate followed by surface drying (5–6 min) in the LAF [23], [25]. Cfu enumeration was done after 18–48 h with the marking of colonies on the reverse of the plate.

The colony development pattern at different dilutions in SP-SDS was recorded as spot growth, too many to count (tmtc) or countable/acceptable (6–60 range). After recording the dilution level yielding acceptable colonies and the cfu per sector, cfu per 100 μl was worked out as *n* × 5 (*n* = colonies in 20 μl sample applied area). The cfu ml^−1^ of the 10^0^ stock was arrived at as the product of *n* × 5 × 10^d+1^ (d = dilution level yielding the countable colonies).

### 2.4 Preliminary SP-SDS trials

An initial SP-SDS trial was set up employing serial dilutions of *E. cloacae* and with irrigation grade open-tank water using six decimal dilutions from the 10^0^ stock with four replications. An assessment of the need for sample vortexing to disperse the bacterial cells during serial dilutions was undertaken using *E. coli* and *E. cloacae* practicing vortexing for 10 s spans during decimal serial dilutions.

### 2.5 Assessing intra- and inter-plate variations in SP-SDS employing *E. cloacae* and mix inoculum

To get an estimate of the possible sector to sector variations in a plate or inter-plate variations during SP-SDS, *E. cloacae* serial dilutions of 10^4^ and 10^5^ were spotted (20 μl) in three sectors each in ten fresh NA plates of which 10^4^ yielded tmtc and 10^5^ countable colonies. The cfu per sector (average of three sectors at 10^5^), standard deviation (SD) and coefficient of variation (CV) were worked out for each plate individually. A similar experiment was undertaken employing the mixed inoculum of five organisms (*E. cloacae, B. pumilus, B. thuringiensis,* S*. epidermidis* and *M. esteraromaticum*) which were pooled in equal proportions employing the dilution levels that yielded the acceptable cfu (30–300 per 100 μl).

### 2.6 Assessing the number of replications needed for comparable cfu estimates in SP-SDS and SATS

SP-SDS was undertaken in comparison with SATS using *E. cloacae* 10^5^ dilution. SATS involved 100 μl sample applied in 12 NA plates while in SP-SDS, 10^5^ dilution was applied in six sectors in 12 plates. Colony counts were made adopting one sector per plate sequentially representing the six sectors across 12 plates in SP-SDS and the cfu/100 μl was recorded in both methods. The mean, SD and CV were worked out sequentially for 2–12 replications. Further, the data were tested for significance through single factor ANOVA considering two to 12 replications sequentially. ANOVA between SATS and SP-SDS was also done considering the average cfu counts from the six sectors in the 12 SP-SDS plates. The experiment was repeated employing a composite sample comprising of *E. cloacae, B. pumilus, B. thuringiensis, S. epidermidis* and *M. esteraromaticum* prepared as above but in irrigation grade tank water with a prior SP-SDS assessment of cfu to fix the appropriate dilution level.

### 2.7 Testing SP-SDS versus SATS on additional pure cultures and composite samples

The applicability of SP-SDS was tested employing pure cultures of different bacteria. This included *E. coli, E. cloacae, P. aeruginosa, A. junii*, *B. pumilus, B. subtilis, B. thuringiensis, S. epidermidis,* S*. haemolyticus* and *M. esteraromaticum* employing NA at 30 °C except for *E. coli* for which trypticase soy agar (TSA; 37 °C) was used. Employing d1–d2 source cultures, 0.1 OD (10^0^) stocks were prepared in FDW followed by the preparation and usage of six decimal serial dilutions. A similar experiment was undertaken with the yeast strain on PDA. The composite samples included irrigation-grade tank water, milk, ground mixed-vegetables and a soil sample. For pure bacterial cultures, the tested dilutions included 10^1^–10^6^, for clear water 10^0^–10^5^, for milk, ground vegetables and soil, 10^1^–10^6^ avoiding the particulate 10^0^. Cfu enumeration was done manually after 18–48 h and beyond as needed depending on the organism/sample.

Based on the information of the appropriate decimal dilution that yielded 30–300 cfu per 100 μl sample, SP-SDS was undertaken in comparison with SATS employing four replicate plates for SATS and adopting cfu from first four of the six sectors in an SP-SDS plate for statistical analyses. Two independent serial dilutions were prepared each applied in duplicate SATS plates or three SP-SDS sectors each. The cfu counts were translated to cfu ml^−1^ of 10^0^ stock and analyzed for significance employing single factor ANOVA (Microsoft Excel 2010) after logarithmic transformation.

### 2.8 Comparison of SP-SDS with alternate resource saving approaches

A comparison of SP-SDS with alternate resource saving techniques was undertaken employing pure cultures of *E. cloacae*, *B.* *pumilu*s, *S. epidermidis* and irrigation grade water. This included 6 × 6 drop-plating as per [2] and track-dilution as per [13]. For track dilution, 12 × 12 cm plates from M/s. HiMedia BioSciences, Mumbai were employed.

### 2.9 Testing SP-SDS approach across other media and NA plates of different batches

SP-SDS approach was tested across other media including Luria Bertani agar for *E. coli*, plate count agar, brain heart infusion agar, Muller Hinton agar and MacConkey agar for *E. cloacae* and irrigation grade water. CNA medium was tested employing *P. aeruginosa*. The media formulations were sourced from M/s HiMedia Biosciences, Mumbai.

Based on the earlier documentations that the quantity of medium per plate, the age of plates after the preparation and the pre-treatments given to the plates did not alter the cfu estimates in SATS [23], fresh plates with 15, 20 or 30 ml NA were tested in SP-SDS for the time needed for droplet drying and the cfu after applying the 10^5^ dilutions of *E. cloacae* and *B. pumilus*. Further, 20 ml NA plates prepared on the same day or that prepared 1–7 days before and the plates given a 37 °C pre-warming treatment were tried in SP-SDS wherein fresh 20 ml NA plates served as control. The experiments were repeated wherein *B. pumilus* culture was employed at a non-decimal dilution (1:3 of 10^4^) to get more acceptable cfu range (>100 per 100 μl) as in the earlier study [25].

### 2.10 Testing SP-SDS methodology employing multi-well plates

SP-SDS methodology was tested with *E. cloacae* and *B. pumilus* using 96 cavity (500 μl) autoclavable polypropylene assay plates (Cat. No. P.96-450R-C; Genaxy Scientific Pvt., Ltd., Solan, India) for serial dilutions adopting 40–400 μl or 50–500 μl decimal dilutions (10^1^–10^6^). As controls 100–1000 μl and 40–400 μl dilutions in 1.5 ml microfuge tubes were employed. Additionally, ELISA plates (Greiner Bio-One GmbH, Germany) were tried which accommodated 200 μl sample per well employing 20–200 μl dilution series.

### 2.11 Testing of SP-SDS methodology for microbiological and biotechnological samples

Different samples representing biotechnology, agriculture, medicine, food microbiology, environmental microbiology and applied microbiology where there was no clear idea about the prevalent bacterial or yeast cfu in the sample were tested through the SP-SDS approach. The preferred dilutions from the anchored stocks included 10^0^–10^5^ or 10^1^–10^6^ for liquid samples, and 10^1^–10^6^ for solid samples avoiding the particulate 10^0^. Further, SP-SDS was tried for parallel testing of two or multiple samples in a plate. This included testing the effect due to different diluents on *E. cloacae* where the 10^0^ stock in FDW was taken through serial dilution in saline (NaCl 9 g l^−1^), phosphate buffered saline (PBS), peptone–water (10 g l^−1^ peptone and 5 g l^−1^ NaCl; pH 7.2) [1], peptone-salt (1 g l^−1^ each peptone and NaCl; pH 7.0; [23]) or nutrient broth (pH 7.4) employing FDW as control. In another trial, *E. cloacae* dilutions prepared in FDW and peptone salt was monitored with SP–SDS after static incubation over 5 h at 20 min intervals during the initial one hour and hourly thereafter employing the decimal dilutions 10^3^–10^8^. Further experimental details are provided under Results and Discussion.

### 2.12 Statistical analysis

For direct comparisons within SP-SDS trials, the mean colony counts per sector in a plate and for comparisons between SP-SDS and SATS techniques, cfu per 100 μl samples were used for statistical analysis. The mean, SD and CV were employed for direct comparisons estimated with the Σ function in Microsoft Excel 2010. In the trial comparing SP-SDS *versus* SATS, the significance was tested through single factor ANOVA or Student’s *t*-test using the Data Analysis Tool of Microsoft Excel 2010 after logarithmic transformation of cfu for the 10^0^ stocks. Unless mentioned differently, four replications were employed for comparing SP-SDS versus SATS.

## 3 Results and discussion

### 3.1 Preliminary SP-SDS trials

**Fig. 2:**
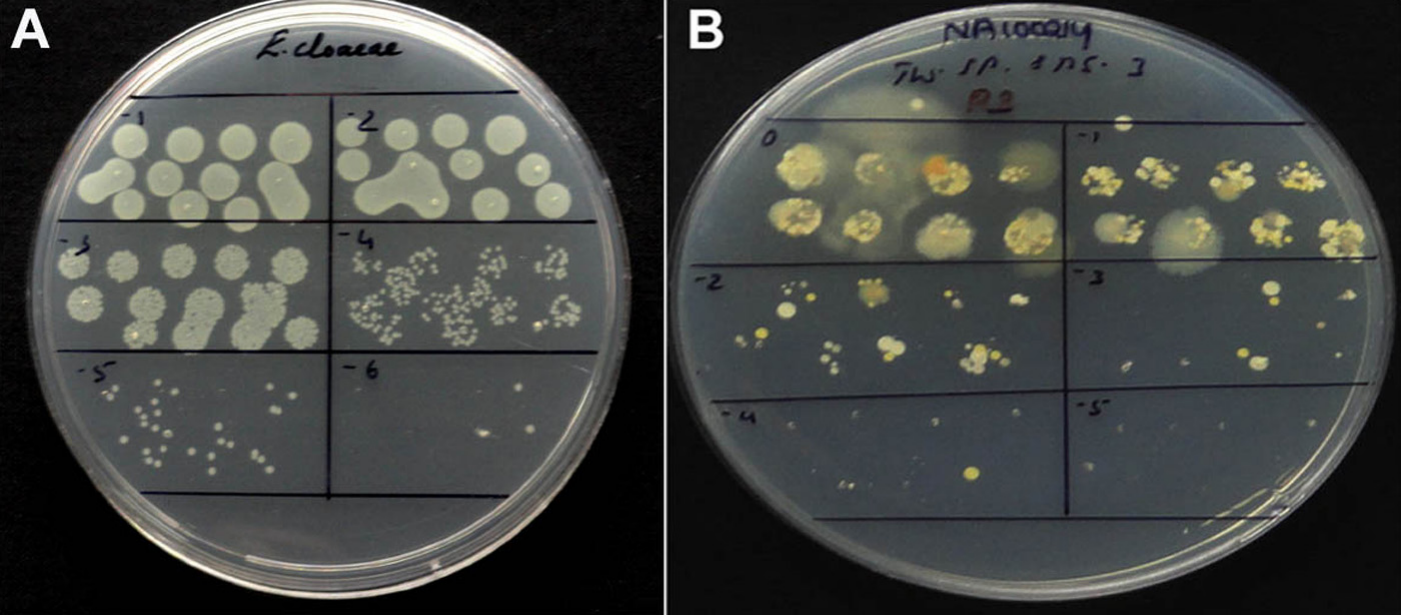
SP-SDS with *Enterobacter cloacae* involving 10^1^–10^6^ dilutions showing acceptable cfu at 10^5^ (A); SP-SDS with static water sample from an open tank at 10^0^–10^5^ dilutions displaying countable colonies at 10^3^ dilution (B).

In the initial trial employing *E. cloacae*, the first three serial dilutions (10^1^–10^3^) showed spot growth, 10^4^ displayed tmtc and 10^5^ yielded well delineated colonies in the acceptable range (Fig. 2A). The plates applied with the irrigation-grade tank-water exhibited cluster of diverse colony types at 10^0^–10^2^ including some spreaders and at 10^3^ countable colonies (Fig. 2B). Thus, at least one dilution in a plate yielded cfu in the acceptable range ensuring the success of the trial. *E. cloacae* displayed full colony emergence by day-1 itself whereas colony development continued for 2–4 days for irrigation water. Marking the initially formed colonies on the reverse of plates helped in identifying late emerging ones and discriminating the fast-growing or spreading colony types. Vortexed and non-vortexed samples of *E. coli* and *E. cloacae* showed similar cfu for both the treatments (data not shown) indicating that vortexing during serial dilutions was not a necessity for homogeneous suspensions but the same did not impart any adverse effect.

### 3.2 Assessing intra- and inter-plate variations during SP-SDS

**Fig. 3:**
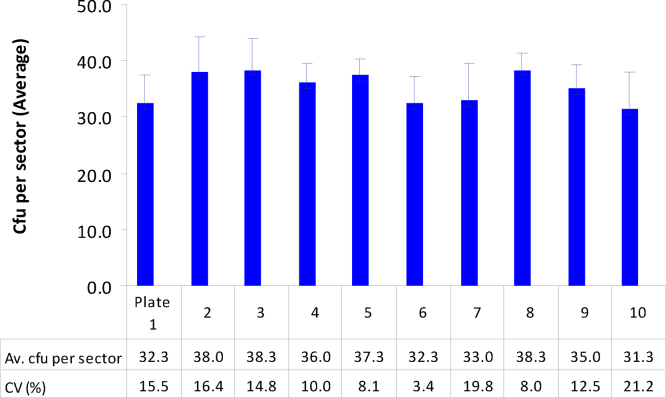
Assessment of intra- and inter-plate variations in cfu during SP-SDS employing *Enterobactercloacae* with the 10^5^ serial dilution from the 0.1 OD stock applied in three sectors in ten NA plates (20 μl/sector). Vertical bars indicate SD per plate.

*E. cloacae* 10^4^ dilution showed tmtc while 10^5^ dilution exhibited cfu in the range of 27–41 per sector with the mean sector cfu of 31.3–38.3 across 10 different plates (Fig. 3). No significant plate to plate cfu variations were observed in the trials employing *E. cloacae* (*P* = 0.553) and the mixed inoculum (*P* = 0.0673). The cfu per sector for the mixed inoculum varied from 19 to 34 across 10 plates and the mean cfu per sector in a plate ranged from 24.0 to 29.8.

### 3.3 Assessment of the number of replications needed for comparable cfu estimates in SP-SDS and SATS

**Fig. 4:**
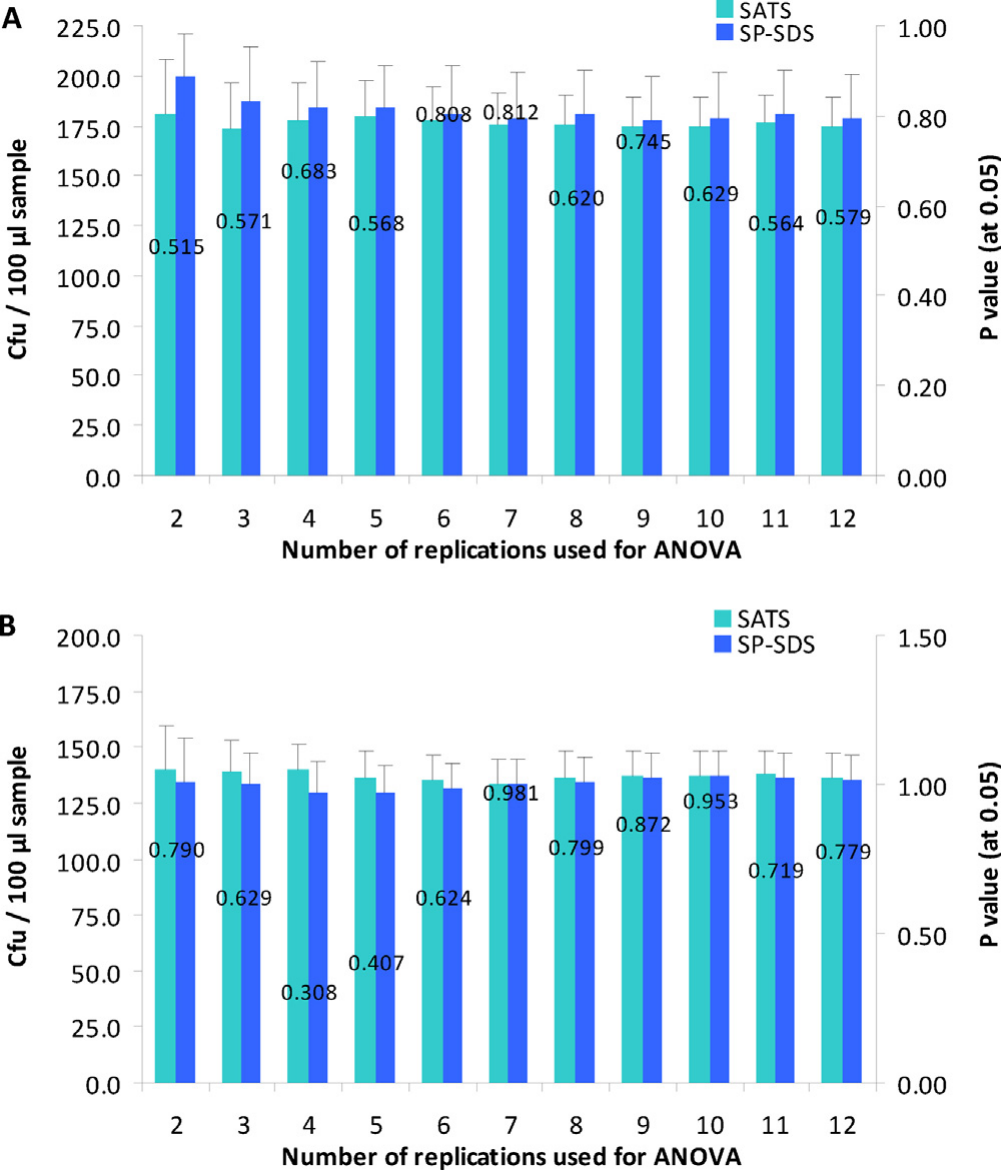
Assessment of the number of replications needed to obtain comparable cfu estimates in SP-SDS and SATS methods using *Enterobacter cloacae* (A) or the mixed inoculum of five organisms in irrigation-grade-water (B) employing 12 replications. Single factor ANOVA was done employing 2–12 replications for cfu per 100 μl sample; Vertical bars indicate SD and the values over the bars indicate the *P* (0.05) value from ANOVA.

The ANOVA results with *E. cloacae* employing 2–12 replications indicated statistically comparable cfu for SATS and SP-SDS (*P* > 0.05 in all instances) starting with two replications (Fig. 4A). The same appeared true for the mixed inoculum prepared in irrigation-grade water (Fig. 4B). Based on these results and the observations from the subsequent trials, use of four replications for SP-SDS was fixed to give similar cfu as in SATS. No definite advantage of using >4 replications was observed based on mean, SD and CV for *E. cloacae* and for the mixed inoculum.

### 3.4 Testing SP-SDS versus SATS on additional organisms and composite samples

Adopting SP-SDS with pure bacterial cultures, most of the colony development occurred within 18–24 h and within 2 days for slow growing organisms like *M. esteraromaticum* and *A. junii*. The 10^0^–10^2^ dilutions from the anchored stocks often displayed spot growth, 10^3^–10^4^ tmtc and 10^3^–10^6^ isolated colonies depending on the organism apparently governed by the cell size as per the earlier report [25]. For instance, *B. thuringiensis* with large rods and *S. epidermidis* with large cocci showed countable cfu at 10^4^ dilution; *E. coli*, *E. cloacae, P. aeruginosa, B. pumilus, B. subtilis* and *M. esteraromaticum* with medium-size rods or *S. haemolyticus* with smaller cocci at 10^5^*,* and *A. junii* with very small cells at 10^6^ (Table 1). This was confirmed in repeat trials when *M. esteraromaticum* and *A. junii* showed some variations with the acceptable cfu at 10^5^ or 10^6^ in some trials. As for *Bacillus* spores, the dilution level for acceptable cfu also varied with the organism (10^3^–10^4^) depending on spore size as documented earlier [24], [26]. This applied to pure yeast culture too (10^3^). The composite samples- mixed inoculum, water, milk, food articles and soil samples- also showed spot growth, tmtc or acceptable cfu depending on the dilution level. Nearly 80–90% colonies emerged within 1–2 days but it required 3–4 days to allow most colonies to develop. Spreaders were occasionally encountered for water, milk and soil samples, particularly with incubation beyond 3–4 days. This applied equally to SATS, spread-plating and pour-plating (Thomas, unpublished results).

SP-SDS worked satisfactorily for all test organisms and composite samples giving acceptable cfu at least in one of the six dilutions from the anchored stocks in a plate. Now, comparing SP-SDS with SATS, the mean cfu recorded for the 10^0^ stocks of pure bacterial cultures or spores in different organisms, yeast and in composite samples over four replications appeared statistically on par (Table 1). The SD recorded in SP-SDS was higher in all instances (average cfu-SD for the 19 samples listed in Table 1, in SP-SDS and SATS, 1.44 × 10^7^ and 9.3 × 10^6^, respectively) but the means from four replications appeared close to each other. Thus, SP-SDS with the anchored stocks offered assured and comparable results to SATS with mere four nutrient plates as against 24 plates needed for a similar testing through conventional plating approaches.

For organisms such as *B. pumilus, B. subtilis, B. thuringiensis* and *A. junii*, the cfu registered for the selected decimal dilution was close to the lower acceptable range while the previous decimal dilution level showed tmtc. This was influenced by the cell size, which is a characteristic feature of the organism. In such instances, the adoption of non-decimal dilutions (*e.g.* 1:3 of 10^4^ for *B. pumilus* and *B. subtilis*) were tried for clearer results which showed identical cfu in SATS and SP-SDS (data not shown).

### 3.5 Comparison of SP-SDS with alternate resource saving approaches

**Fig. 5:**
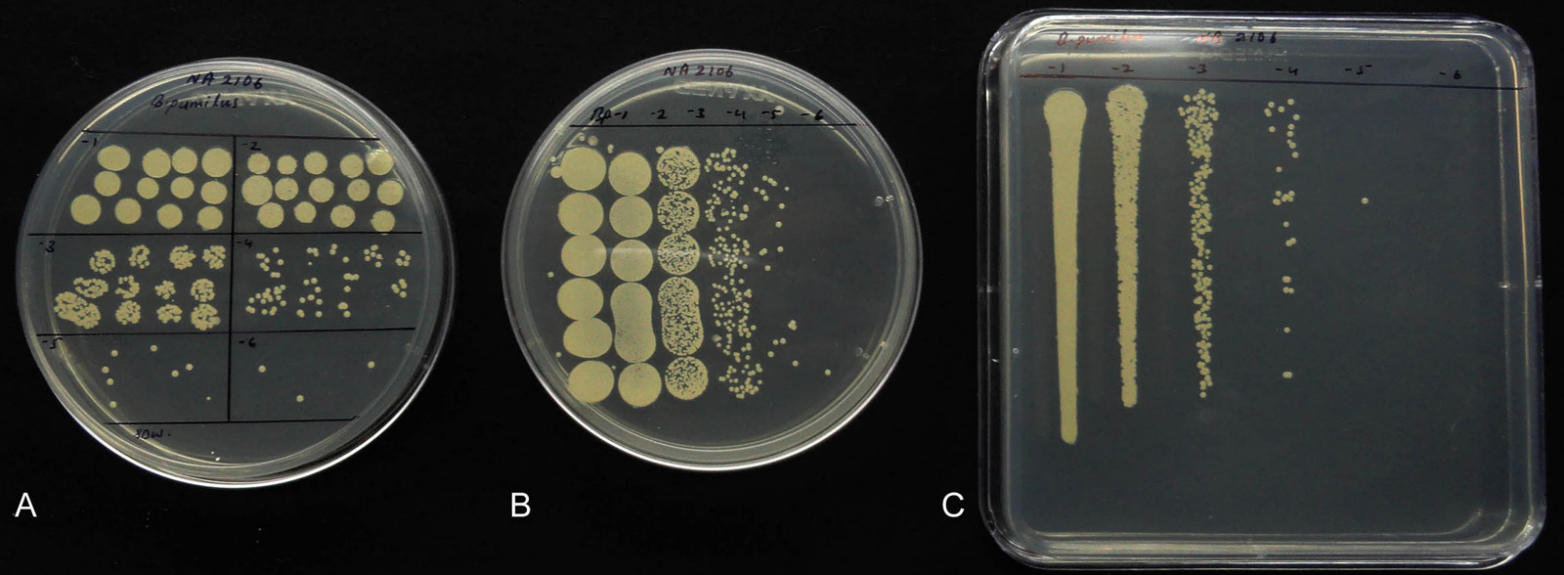
Comparison of SP-SDS (A) with 6 × 6 drop-plating (B) and track-dilution (C) at the serial dilutions of 10^1^–10^6^ for *Bacillus pumilus* showing acceptable cfu in SP-SDS at 10^5^ dilution.

A comparison of SP-SDS with 6 × 6 drop-plating and track-dilution indicated that the former be advantageous over the two other methods in several respects (Fig. 5; Table 2). The 6 × 6 drop-plating was fine for slow growing pure cultures but not for fast growing colony types and for composite samples that bore organisms differing in colony growth rates. SP-SDS worked well for all samples including pure cultures, spores and composite/environmental samples. This also applied to track dilution but for the high cost of square plates.

### 3.6 Testing SP-SDS method across other media and NA plates of different batches

SP-SDS approach with decimal dilutions worked well across different media yielding well delineated colonies for pure cultures of most of the organisms. *B. subtilis* and *B. thuringiensis* showed tendency for fast or spreading colony development. Testing NA plates with different amounts of medium, *E. cloacae* showed identical cfu in plates with 15, 20 or 30 ml fresh medium (50.8, 51.0 and 53.3 per sector at 10^5^ dilution, respectively; NS). The same appeared true for *B. pumilus* (11, 11.5 and 11 cfu per sector, respectively, for 10^5^ stock and 28.6, 27.0 and 24.0, respectively in the trial employing 1:3 dilution of 10^4^ stock; NS). This indicated considerable saving of media resources with the use of 15–20 ml medium per plate. Besides, the time needed for the droplet drying was considerably shortened with the reduction in the amount of medium per plate (4, 6 and 10 min, respectively for 15, 20 and 30 ml fresh NA plates). The colonies in 15–20 ml appeared smaller and stayed confined for longer time than in 30 ml plates.

**Fig. 6:**
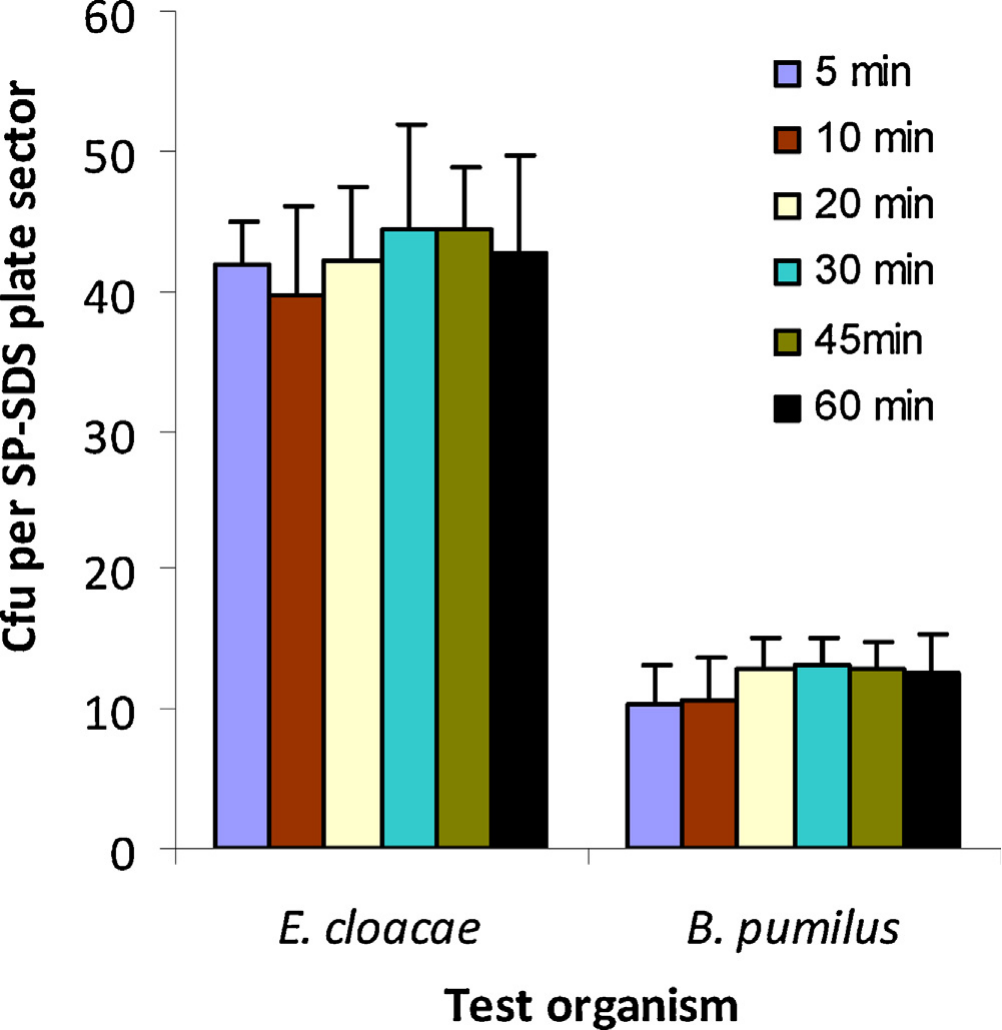
Effect due to the extended open-plate drying in the laminar airflow cabinet for 5–60 min on cfu per SP-SDS plate sector in *Enterobacter cloacae* and *Bacillus pumilus.*

One stipulation with SP-SDS was the proper drying of droplets in the LAF cabinet. Testing fresh 20 ml NA plates *versus* media prepared 1–7 days before (sealed in PP bags), or refrigerated for a month, the cfu estimates for *E. cloacae* were unaltered between them, but the older plates offered considerable saving of time towards sample drying. The time for droplet drying was governed by the free moisture content in the medium. For instance, freshly poured NA plates (20 ml) used within 1 h required about 6–8 min which was reduced to 4–5 min by 2 h after pouring and to about 1–2 min if used after one or a few days after preparation. Pre-warming the plates at 37 °C was not a necessity nor offered any advantage but often caused water condensation which ought to be removed before using the plates. Variable durations of open plate incubation in the LAF did not alter the cfu for up to 60 min as observed with *E. cloacae* and *B. pumilus* cultures (Fig. 6; *P* > 0.05 in both instances) allowing flexibility in the operations. Thus, SP-SDS technique worked fine with freshly prepared plates, previously prepared refrigerated or ambient stored plates (sealed in PP covers) and even plates with partial dehydration, and also worked well with varying amounts of medium per plate as per the observations employing *E. cloacae* similar to the findings with SATS technique [23].

### 3.7 SP-SDS methodology with multi-well plates

Testing SP-SDS method with the use of 96 cavity assay plates for serial dilutions showed that 40–400 μl dilutions was feasible but not 50–500 μl series due to the chances of inoculum mixing between adjoining wells. Comparing 40–400 μl dilution series in assay plates *versus* 40–400 μl or 100–1000 μl dilution in 1.5 ml microfuge tubes showed similar cfu in the three treatments for *E. cloacae* (39.0 ± 3.93, 44.5 ± 5.54 and 36.5 ± 2.5 cfu per sector, respectively; *P* = 0.108). The corresponding figures for *B. pumilus* were 11.7 ± 5.60, 11.7 ± 2.21 and 14.0 ± 4.54 (*P* = 0.711). ELISA plates were not preferred as they could accommodate only smaller volume (200 μl) besides their high cost and non-feasibility for reuse unlike the autoclavable assay plates.

### 3.8 Demonstrating the applications of SP-SDS in microbiology and biotechnology

**Fig. 7:**
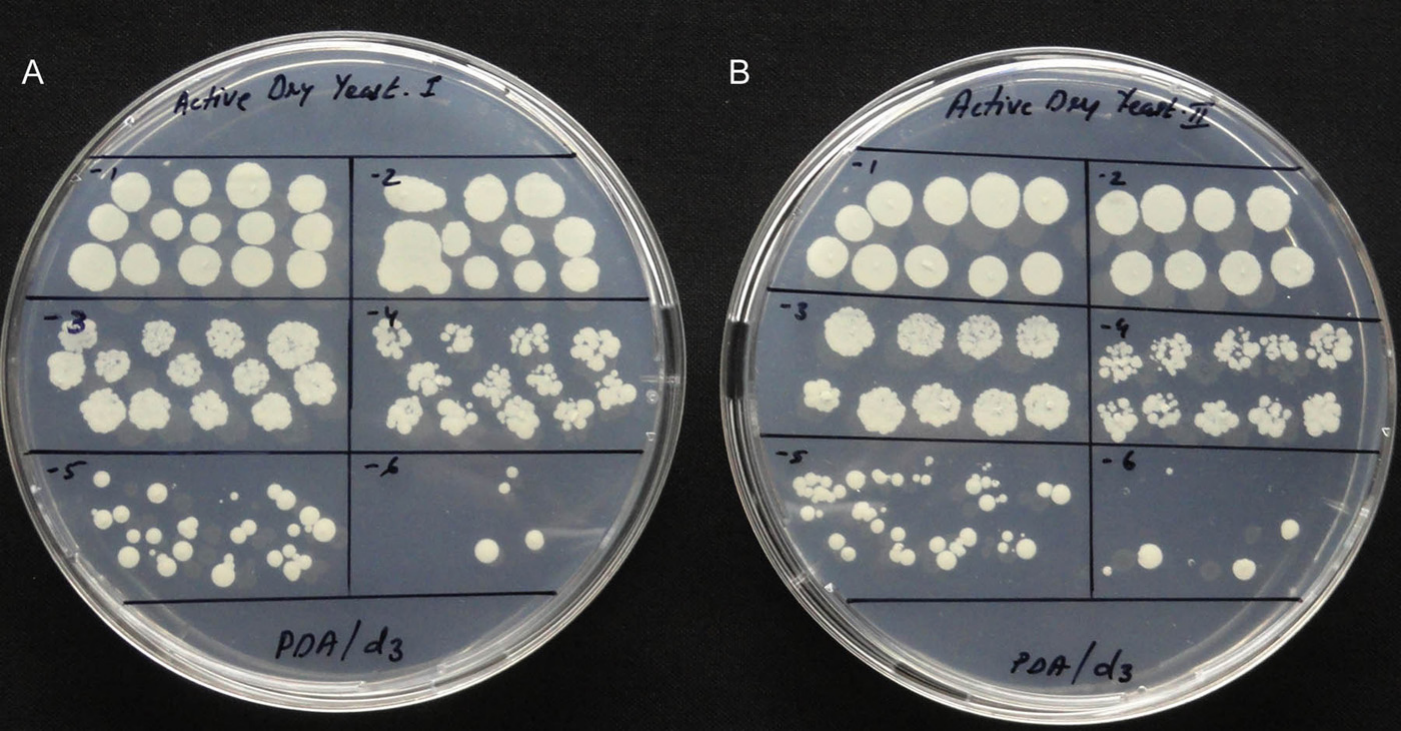
SP-SDS on two market brands of active dry yeast at 10^1^–10^6^ dilutions on PDA showing acceptable cfu at 10^5^ in A and in B on day-3.

SP-SDS method worked well for various samples tested where there was no prior idea of the dilution level that would yield countable cfu giving acceptable colony counts for at least one of the six dilutions contrary to the six plates required in SATS, spread-plating, pour-plating or spiral plating to accommodate same number of dilutions. Besides pure and mix cultures, these included environmental samples, probiotic and agricultural bio-formulations, cultures of different organisms post antibiotic challenge and different food products (Table 3). For food samples and other instances where the viable counts or the microbial composition would change with time or storage, SP-SDS formed an ideal tool for cfu assessment. Use of 4–6 replicate plates is recommended for testing such items that could not be stored or retested. The utility of SP-SDS technique was noteworthy while testing broth cultures in different stages of growth or an organism grown under different conditions, testing the effect due to antibiotics and other antimicrobials on single organisms or mix-cultures where the extent of cfu reduction varied depending on the organism and the chemical employed. Water and soil samples introduced with clinically significant *P. aeruginosa* (*Pau*) could be specifically monitored for *Pau* on CNA selective medium with parallel testing on NA to assess its load and the interactive or inhibitory effects on other microflora. The distinct green-tinge of fluorescent *Pau* colonies allowed their clear identification on NA yielding similar counts on CNA medium [Thomas and Sekhar, unpublished results]. The SP-SDS method also worked satisfactorily for market lots of active dry yeast yielding delineated colonies on PDA (Fig. 7). While pure yeast culture displayed more or less uniform colony emergence, the market lots showed colony development spanned over 2–3 days.

### 3.9 Significance of SP-SDS methodology and further optimizations

**Fig. 8:**
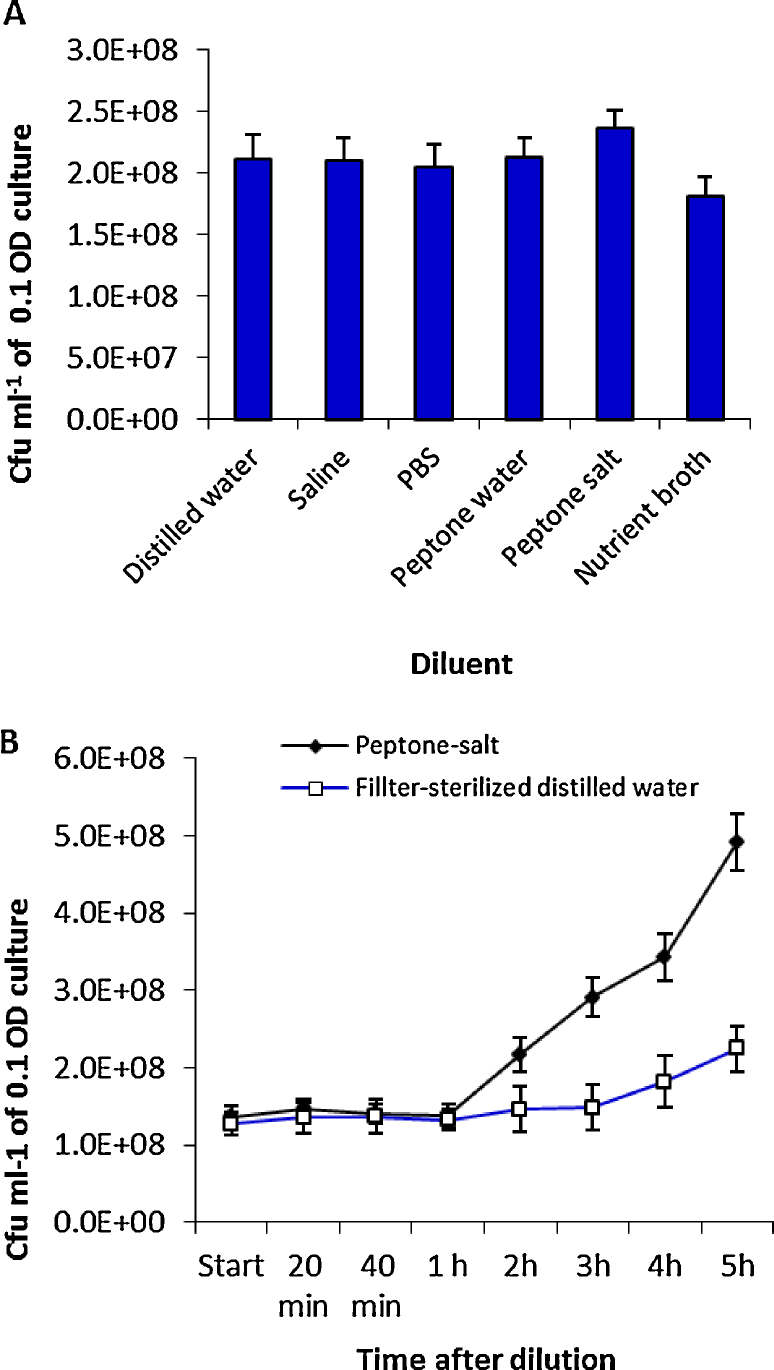
Testing the effect due to the diluent on cfu employing *Enterobacter cloacae* by diluting the 10^0^ water stock in distilled water, saline, PBS, peptone–water, peptone–salt or nutrient broth (A) and monitoring *E. cloacae* 10^5^ dilution prepared in filter sterilized distilled water or peptone-salt over 5 h static incubation for bacterial multiplication through cfu estimation (B). Vertical bars indicate SD.

The hall mark of SP-SDS was ensuring acceptable cfu at one of the decimal dilutions thereby safeguarding against the failure of cfu assessing trials. SP-SDS was particularly useful for side by side testing of two or multiple samples. For instance, we were eager to determine the most appropriate diluent without adverse or contributory effects on cfu due to bacterial cell lysis or multiplication during the SP-SDS procedure. Testing six different diluents for *E. cloacae* which could be done by accommodating the six treatments in a single plate for their direct comparisons, the maximum cfu was recorded for peptone-salt followed by FDW, saline, PBS and peptone-water (on par) while NB registered a notably lower cfu (Fig. 8A). To ascertain the lower colony counts in NB, we further tested NB in comparison with FDW and peptone-salt (PS) as controls on *E. cloacae* and *B. pumilus*. This again indicated a lower cfu with NB in *E. cloacae* (46.8, 39.6 and 37.0 cfu per sector for FDW, PS and NB, respectively) as well as for *B. pumilus* (12.0, 8.8 and 8.3, respectively, using 10^5^ dilution, and 34.2, 31.2 and 18.5, respectively, for 1:3 dilution of 10^4^). The undesirable NB effect appeared to arise from the inhibition to cell germination due to higher nutrient levels at the sample dried spots. Further, testing the growth of the two organisms in 1.0× and 1.25× NB with overnight shake incubation indicated that the growth was not enhanced but rather reduced at higher NB level in both *E. cloacae* (OD600 nm of 1.696 and 1.346, respectively; *P* = 0.028) and *B. pumilus* (1.212 and 1.033, respectively; *P* = 0.001). Thus, it appeared that NB was not a preferred diluent while FDW and PS appeared fine.

Further, we made a comparative assessment between FDW and PS to test the cell stability in FDW *vis-à-vis* the possibility of bacterial multiplication in PS during the course of SP-SDS procedure employing *E. cloacae.* For this, the 1.0 OD bacterial suspension prepared in FDW was diluted to 10^5^ in PS or FDW and monitored over an extended period at 20 min intervals for 1 h and hourly thereafter for 5 h. This showed comparable cfu for up to 2 h in FDW and a slow increase thereafter. PS showed similar cfu during the first 60 min, but a significant increase between 1 and 2 h (Fig. 8B). These observations endorsed the usage of FDW as the preferred diluent. In the event of using enriched diluents, it warranted that the samples be refrigerated/chilled on ice with minimum time between sample preparation and deposition. SP-SDS approach also proved advantageous in monitoring various situations of ambiguous viable counts by accommodating multiple dilutions in a plate. It also served as a pre-trial to fix the dilution levels for sample analysis through SATS as in our surveillance of *P. aeruginosa* introduced in water or soil.

**Fig. 9:**
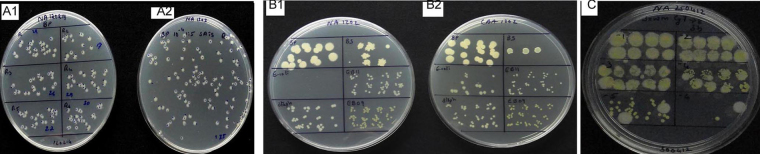
SP-SDS applications in microbiology and biotechnology: Feasibility of accommodating up to six replications of a selected dilution in a single plate in SP-SDS (A1) in comparison with one replication per plate in SATS (A2); Parallel testing of six different organisms (left to right from top: *Bacillus pumilus*, *B. subtilis, Escherichia coli, Enterobacter cloacae, Staphylococcus epidermidis* and *Microbacterium esteraromaticum*) on nutrient agar (B1) *versus* Luria Bertani agar (B2) with *E. coli* showing delayed growth on NA at 30 °C; SP-SDS showing culture admixture where the contaminant appears as large colonies at 10^5^ and 10^6^ dilutions (C).

When a known dilution level was to be tested, SP-SDS offered the advantage of six replications in a plate as against six separate plates needed in SATS (Fig. 9A). It facilitated the parallel testing of 2, 3 or 6 samples under uniform conditions in a plate (Fig. 9B). Presentation of different dilutions side by side in a plate over an extended area allowed easy detection of mixtures and culture purity testing (Fig. 9C) which forms an essential requirement during microbe-microbe interaction studies and molecular investigations. SP-SDS formed a very ideal tool for single colony purification from mixed stocks with one or more dilutions yielding distinct single colonies.

Most of the environmental specimens employed in this study showed acceptable cfu within the first four dilutions. Applying four dilutions (10^1^–10^4^) in four sectors per plate allowed more area per sample accommodating diverse and even spreading colony types. No differences in cfu per sector were observed if four or six sectors were prepared in a 9-cm plate as observed with *E. cloacae* (42.6 ± 7.55 and 47.6 ± 6.91, respectively) and *B. pumilus* (13.3 ± 3.32 and 10.8 ± 3.48, respectively). When diversity analysis was the objective, running a pre-trial with SP-SDS helped in identifying the preferred dilution based on which SATS trials could be set up to cover low abundant types.

Although the usage of known sample weight per unit volume for solid food articles or the use of direct samples for liquid specimens as starting stock is a common practice in microbiology, none of the publications specifically emphasize the need for ‘sample anchoring’ as a standard practice. Publications addressing cfu monitoring in pure cultures often use serial dilutions of bacterial suspensions or broths and report final growth assessments based on cfu and OD rather than anchoring the OD initially. The concept of accommodating multiple serial dilutions in a nutrient plate is also in vogue in bacteriology [2], [13]. The significant aspects of this study have been the prescription of sample anchoring to a specific and reference base (10^0^ stocks) as a standard practice at the start of the trial plus the accommodating multiple dilutions in a plate. Anchoring the specimens ensured that at least one dilution level yielded acceptable colony counts in a plate and that the experiment would not fail wholly in the absence of which some trials overshot the acceptable cfu level in a plate. With the identification that the cfu ml^−1^ in an organism at a particular OD is governed by cell or spore size [23], [24], [25], [26], we are now able to set up SATS trials with most organisms at the dilutions mentioned in Table 1. In some instances, the non-decimal dilutions were needed to obtain a higher cfu (>100 per 100 μl) in critical comparative trials as documented earlier [25], [26]. It was essential that a relatively thin suspension of 0.1–0.5 OD be used for the OD estimation for precision. For colloidal and thick suspensions, such as milk and juices, the original specimen could be employed directly or after adjusting the OD to a desired level. For instance, milk showed variations in OD600 nm from 150 to over 200 whether it was full cream, toned or skimmed and with brands. Sample anchoring held good also for other modes of cfu estimation such as SATS, spread-plating or pour-plating.

Cfu ml^−1^ in an organism showed some variations with the source culture medium, age of culture or the way a culture was grown which in turn was attributable to differences in cell size [25] or factors such as cell debris or pigments that alter the OD. For instance, in the results presented herein, *E. cloacae* derived from spot-growths showed cfu in the range of 40–50 per sector while that derived from isolated single colonies with larger cells yielded cfu in the 30–40 range. SP-SDS accommodated all such situations with uncertain initial cfu. The major time investment during cfu estimations was preparing the dilution series which applied equally to SP-SDS, SATS, spread-plating and pour-plating. We are not addressing sampling procedures in this study for which the accepted standard procedures prescribed such as International Commission for the Microbiological Specifications of Foods (ICMSF) or International Organization for Standards (ISO) are to be adhered.

Refining the SP-SDS approach further, up to eight dilutions per plate, more amount of sample (25–50 μl) per sector or more area per dilution as for environmental samples and food articles could be accommodated with the use of 10 cm diameter or 12 × 12 cm square plates. It was important that the decimal dilutions show a clear reduction in cfu with dilution series and that the extinction point (no colonies) is attained within the 10^5^–10^7^ dilution in the case of pure cultures absence of which indicated improper serial dilution. This was often noticed when tip-flushing and tip-change during onward serial dilution were not adhered to. This also occurred due to the presence of contaminants in the diluent which could occur due to improper sterilization or their accidental introduction during sample handling reinforcing the need for testing the diluent in each plate. Use of FDW is prescribed as the standard diluent without much adverse effects of cell lysis or bacterial multiplication during the course of SP-SDS procedure. It is not proper to use previously prepared and stored stock cultures or dilutions as the organisms display microaerophlic growth even under refrigeration. As a step to automation, it is possible to capture the plate images and effect the colony counts later on. Thus, SP-SDS appeared advantageous and applicable across different spheres of microbiology and biotechnology for samples of uncertain cfu and under low resource settings. Further, when there is a clear idea of the dilution level for acceptable cfu or for critical comparative trials, we still adopt SATS.

## 4 Conclusions

SP-SDS where six different dilutions of a bacterial suspension or test sample (20 μl) is spotted as micro-drops across a 9-cm plate agar-surface represents a simple, efficient and resource-saving technique for bacterial cfu estimations when there is no clear idea about the initial cfu or the dilution at which countable colonies could be expected. Sample anchoring (use of 10^0^ stock) which in the case of pure bacterial cultures formed the 0.1 OD stock, the original suspension for water and other liquid samples, and 10% (w/v) sample for food and soil specimens, followed by the application of decimal serial dilutions in sterile distilled water ensured that at least one of the dilutions yielded countable colonies in the acceptable range in each nutrient plate. SP-SDS with four replications suited diverse samples including pure bacterial and yeast cultures, spores, mix-bacterial inoculum, food, clinical, environmental and other biotechnological samples giving similar cfu estimates as the standard SATS approach employing 100 μl samples per plate. Besides cfu enumeration, SP-SDS enabled single colony selection and culture purity confirmation.

## Competing interests

The authors have no competing interests.

## Figures and Tables

**Table 1:** Comparison of SP-SDS *versus* SATS employing pure cultures of different organisms and composite samples.

No.	Organism/experimental sample	Working stock dilution for countable cfu	SP-SDS			SATS		Cfu ml^−1^ of 10^0^ stock			Significance
			Cfu range/20 μl	Cfu range/100 μl	Cfu average/100 μl	Cfu range/100 μl	Cfu average/100 μl	SP-SDS	SATS	*P* (0.05)^b^	
	Pure bacterial cultures (day 1 NA/NB stocks)										
1	*Escherichia coli*	10^5^	20–31	100–155	125.0	96–127	110.0	1.25 × 10^8^	1.10 × 10^8^	0.265	NS
2	*Enterobacter cloacae*	10^5^	32–40	160–200	176.25	160–190	170.25	1.76 × 10^8^	1.70 × 10^8^	0.603	NS
3	*Pseudomonas aeruginosa*	10^5^	31–42	155–210	178.75	168–195	183.75	1.79 × 10^8^	1.84 × 10^8^	0.671	NS
4	*Bacillus pumilus*	10^5^	13–18	65–90	76.25	65–78	71.25	7.63 × 10^7^	7.13 × 10^7^	0.474	NS
5	*Bacillus subtilis*	10^5^	11–18	55–90	62.5	34–51	41.5	6.25 × 10^7^	4.15 × 10^7^	0.474	NS
6	*Bacillus thuringiensis*	10^4^	8–16	40–80	63.75	40–63	50.75	6.38 × 10^6^	5.08 × 10^6^	0.291	NS
7	*Staphylococcus epidermidis*	10^4^	33–43	165–215	192.5	157–197	173.8	1.93 × 10^7^	1.74 × 10^7^	0.228	NS
8	*Staphylococcus haemolyticus*	10^5^	30–37	150–185	163.8	158–198	185.5	1.64 × 10^8^	1.86 × 10^8^	0.128	NS
9	*Microbacterium esteraromaticum*	10^5^	46–58	230–290	261.5	214–227	214.75	2.61 × 10^8^	2.15 × 10^8^	0.081	NS
10	*Acinetobacter junii*	10^6^	4–9	20–45	33.75	23–38	27.0	3.38 × 10^8^	2.70 × 10^8^	0.391	NS
11	*Bacillus subtilis* spores	10^4^	48–60	240–300	272.5	264–296	279.25	2.73 × 10^7^	2.79 × 10^7^	0.625	NS
12	*Bacillus thuringiensis* spores	10^3^	46–61	245–305	262.5	256–298	272.75	2.62 × 10^6^	2.73 × 10^6^	0.453	NS

	Composite samples										
1	Mix culture- pooled stocks^a^	PD	14–31	70–155	101.25	81–129	106.0	1.01 × 10^7^	1.06 × 10^7^	0.702	NS
2	Irrigation grade tank water	10^3^	20–35	100–175	142.5	123–141	145.5	1.43 × 10^6^	1.46 × 10^6^	0.793	NS
3	Milk- freshly boiled	10^0^–10^5^	None	None		None		0	0	–	
4	Milk- 6h open incubated	10^2^	15–20	75–100	87.5	83–97	88.5	8.75 × 10^4^	8.85 × 10^4^	0.833	NS
5	Cut mixed-vegetables	10^1^	19–32	95–160	118.75	88–111	100.8	1.19 × 10^4^	1.01 × 10^4^	0.294	NS
6	Rhizospheric soil of banana	10^2^	35–47	175–235	207.5	165–211	183.0	2.08 × 10^5^	1.83 × 10^5^	0.203	NS

	Wine yeast										
1	*Saccharomyces cerevisiae*	10^3^	41–52	205–260	227.5	211–232	222.25	2.28 × 10^6^	2.22 × 10^6^	0.731	NS

Nutrient agar (NA) formed the test medium in all instances except for *E. coli* and yeast for which trypticase soy agar and PDA, respectively, were employed.

a Mix culture: Pooled working stocks of *E. cloacae*, *B. pumilus*, *B. thuringiensis, S. epidermidis* and *M*. *esteraromaticum* in equal proportions and cfu pre-determined (PD).

b ANOVA after logarithmic transformation; NS, not significant.

**Table 2:** Comparative assessment of SP-SDS with other resource saving 6 × 6 drop plating and track dilution methods and the standard SATS method for pure cultures, mix inoculum, spores and composite food and environmental samples.

Attribute	Standard method–SATS^a^	Resource saving methods		
		SP-SDS	6 × 6 Drop-plating^b^	Track-dilution^c^
Plate type and media requirement per plate	9 cm round/ 15–20 ml	9 cm round/15–20 ml	9 cm round/15–20 ml	10 × 10 cm square/25–30 ml
Number of sample dilutions per plate and sample volume	1; 100 μl	6; 20 μl	6; 10 μl	6; 10 μl
Sample application procedure	Spotting as 10–15 drops, tilt spreading and drying for 5–6 min	10–12 micro-drops of ∼2 μl and drying for 5–6 min	Spotting as one drop and drying for 12–15 min	Spotting as drop, plate tipping and drying 30s-1 min
Area available per sample or dilution (approx.)	63.6 cm^2^ (full plate)	9 cm^2^ (one sector)	0.785 cm^2^ one drop of ∼1 cm dia.)	Variable; 10 cm track
No. of replications accommodated per plate	One sixth of SP-SDS	One	Six	One
Sample anchoring to 10^0^ and repeatability	Yes	Yes	No	No
Suitability for pure bacterial cultures	Yes	Yes	Excluding swarming types	Yes
Suitability for spores	Yes	Yes	No mention	No mention
Suitability for mixed bacterial cultures & environmental samples	Yes	Yes	No mention	No mention
Flexibility with agar plates and media	Diverse media; fresh and old; Proper drying post-spotting	Diverse media; fresh and old; Proper drying post-spotting	Properly surface dried LBA, BHIA, MHA plates	Properly surface dried BHIA plates
Relative economic input (cost per sample)	Very high	Medium cost; Easily done with common lab supplies	Low cost	High cost
Other constraints	More plates and incubator space	Manual plate marking	Drop merging; Need for 96-well plates and multi-channel pipette	Track migration

Abbreviations: BHIA, brain heart infusion agar; LBA, luria bertani agar; MHA, Muller-Hinton agar; SATS, Spotting-and-tilt spreading; SP-SDS, Single Plate-Serial Dilution Spotting.

a Refs. [23], [25].

b Ref. [2].

c Ref. [13].

**Table 3:** Demonstration of the utility of SP-SDS for testing diverse samples with uncertain viable bacterial or yeast colony counts.

	Experimental sample/specimen	Anchored stock	Dilutions tested	Dilution yielding cfu	CFU range/sector	Av. cfu/100 μl	cfu ml^−1^ of 10^0^ stock	Remarks
	Monitoring for bacteria on nutrient agar (NA)							
1	Fresh tap water- non potable	Direct	10^0^–10^5^	10^1^	25–30	137.5	1.38 × 10^4^	
2	Mini-aquarium fresh water	Direct	10^0^–10^5^	10^2^	48–55	258.8	2.58 × 10^5^	
3a	Dry field soil	1 g/10 ml	10^1^–10^6^	10^3^	9–11	50.0	5.00 × 10^5^	Spreaders
3b	Dry soil- moistened overnight	1 g/10 ml	10^1^–10^6^	10^3^	34–59	240.0	2.40 × 10^6^	Spreaders
4	Banana root tissue	1 g/10 ml	10^1^–10^6^	10^1^	10–16	65.0	6.50 × 10^3^	
5	Yakult^®^: Fermented probiotic milk drink	Direct	10^1^–10^6^	10^6^	10–15	60.5	6.05 × 10^8^	Claimed min: 1 × 10^8^
6	Lignite based agricultural consortium	1 g/10 ml	10^1^–10^6^	10^3^	6–13	46.3	4.63 × 10^5^	
7a	Mix culture of various organisms pre-antibiotic challenge	0.1 OD	10^1^–10^6^	10^4^	18–32	145.0	1.45 × 10^7^	
7b	Mix culture of various organisms in antibiotic for 1 h	0.1 OD	10^0^–10^5^	10^2^	11–19	76.7	7.68 × 10^4^	No growth from 10^0^
8a	Bottled pulpy orange juice- fresh	Direct	10^0^–10^5^	–	0	0	0	No growth
8b	Bottled pulpy orange juice-6 h open incubated	Direct	10^0^–10^5^	–	0	0	0	No growth
9	Opened tetra pack fruit juice refrigeration stored for 1 month	Direct	10^0^–10^5^	–	0	0	0	No growth
10	*Pseudomonas aeruginosa* (*Pau*) pure culture on CNA	0.1 OD	10^1^–10^6^	10^5^	31–42	178.8	1.79 × 10^8^	
10a	Tap water added with 100 μl of 0.1 OD *P. aeruginosa* per ml/tested on NA	0.1 OD	10^1^–10^6^	10^4^	45–57	238.75	2.39 × 10^7^	*Pau* + non-*Pau*
10b	-do- tested on CNA	0.1 OD	10^1^–10^6^	10^3^	30–39	1.77	1.78 × 10^6^	Only *Pau*
10c	Soil inoculated with Pau: testing on NA	1 g/10 ml	10^1^–10^6^	10^4^	10–17	6.37	6.38 × 10^6^	*Pau* + non-*Pau*
10d	-do- tested on CNA	1 g/10 ml	10^1^–10^6^	10^1^	16–27	102.5	1.03 × 10^4^	Only *Pau*

	Monitoring for yeast on potato dextrose agar (PDA)							
1	Active dry yeast- Brand 1	1 g/10 ml	10^1^–10^6^	10^5^	39–46	212.5	2.12 × 10^8^	day 3 count; Pure yeast
2	Dry Bakers yeast- Brand 2	1 g/10 ml	10^1^–10^6^	10^5^	37–55	230.0	2.30 × 10^8^	

Medium employed for bacterial cultures was NA in all instances unless mentioned differently; CNA, cetrimide- nalidixic acid-agar selective medium for *Pseudomonas aeruginosa*.

## Appendix A. Supplementary data

A video demonstrating the single plate - serial dilution spotting (SP-SDS) method is available on youtube: https://youtu.be/LEqmWmBVIpA with the caption ‘SP-SDS: A Simple technique for microbial CFU enumeration’.
